# Large variations in all-cause and overdose mortality among >13,000 patients in and out of opioid maintenance treatment in different settings: a comparative registry linkage study

**DOI:** 10.3389/fpubh.2023.1179763

**Published:** 2023-09-22

**Authors:** Roman Gabrhelík, Morten Hesse, Blanka Nechanská, Marte Handal, Viktor Mravčík, Christian Tjagvad, Birgitte Thylstrup, Abdu Kedir Seid, Anne Bukten, Thomas Clausen, Svetlana Skurtveit

**Affiliations:** ^1^First Faculty of Medicine, Department of Addictology, Charles University, Prague, Czechia; ^2^Department of Addictology, General University Hospital in Prague, Prague, Czechia; ^3^Centre for Alcohol and Drug Research, Aarhus University, Aarhus, Denmark; ^4^Norwegian Institute of Public Health, Oslo, Norway; ^5^Norwegian Centre for Addiction Research, University of Oslo, Oslo, Norway

**Keywords:** opioid agonist treatment, opioid maintenance treatment, methadone, buprenorphine, buprenorphine with naloxone, treatment outcomes, mortality, registry-based study

## Abstract

**Background:**

Opioid maintenance treatment (OMT) has the potential to reduce mortality rates substantially. We aimed to compare all-cause and overdose mortality among OMT patients while in or out of OMT in two different countries with different approaches to OMT.

**Methods:**

Two nation-wide, registry-based cohorts were linked by using similar analytical strategies. These included 3,637 male and 1,580 female patients enrolled in OMT in Czechia (years 2000–2019), and 6,387 male and 2,078 female patients enrolled in OMT in Denmark (years 2007–2018). The direct standardization method using the European (EU-27 plus EFTA 2011–2030) Standard was employed to calculate age-standardized rate to weight for age. All-cause and overdose crude mortality rates (CMR) as number of deaths per 1,000 person years (PY) in and out of OMT were calculated for all patients. CMRs were stratified by sex and OMT medication modality (methadone, buprenorphine, and buprenorphine with naloxone).

**Results:**

Age-standardized rate for OMT patients in Czechia and Denmark was 9.7/1,000 PY and 29.8/1,000 PY, respectively. In Czechia, the all-cause CMR was 4.3/1,000 PY in treatment and 10.8/1,000 PY out of treatment. The overdose CMR was 0.5/1,000 PY in treatment and 1.2/1,000 PY out of treatment. In Denmark, the all-cause CMR was 26.6/1,000 PY in treatment and 28.2/1,000 PY out of treatment and the overdose CMR was 7.3/1,000 PY in treatment and 7.0/1,000 PY out of treatment.

**Conclusion:**

Country-specific differences in mortality while in and out of OMT in Czechia and Denmark may be partly explained by different patient characteristics and treatment systems in the two countries. The findings contribute to the public health debate about OMT management and may be of interest to practitioners, policy and decision makers when balancing the safety and accessibility of OMT.

## Introduction

Opioid use disorders (OUD) are linked to elevated mortality rates among users of opioids across regions (1, 2). Mortality among regular illicit opioid (e.g., heroin) users is nearly 15 times higher compared with their peers, with higher mortality during out-of-treatment periods than during treatment (1). This is further accentuated by the opioid crisis that has unfolded and evolved in the U.S. (3) and other countries. Among the causes of mortality, overdose deaths stand out as the foremost factor among people who use extramedical opioids (4) or people in opioid maintenance treatment (OMT) (5).

OMT is the first-line treatment for OUD. Generally, OMT is a well-established treatment approach following international guidelines (6). Despite the overall increase of OMT coverage in European countries over the past decade, differences in coverage among countries remain, e.g., with France exceeding 80% coverage, while Latvia and Romania show suboptimal coverage with only about 10% of high-risk opioid users receiving OMT (7). While some patients receive medication from a general practitioner, specialized OMT clinics that combine medication with psychosocial care may be more suitable for patients who may benefit from more complex services offered (8). The regimen of these services and the prevailing system of OMT varies within each country, across countries, and over time (7).

Over the past few decades, an increasing number of countries, such as Denmark and Norway, have embraced a more inclusive approach to OMT. This approach is characterized by extensive treatment coverage and less stringent criteria for remaining in treatment, which has resulted in higher retention rates (9). A recent comprehensive review on treatment structure by Kourounis et al. (10) addressed how treatment systems differ in terms of barriers, treatment access, demands placed on patients during treatment, and the degree to which patients had an influence on treatment goals as well as the kinds of services they could receive. The authors conclude that low-threshold harm reduction treatment services are linked to better treatment outcomes than high-threshold treatment services that require patients to submit urine tests, to attend counseling, and are generally more inflexible (10).

There is evidence from both experimental and observational studies that OMT contributes to the reduction in mortality rates among patients in OMT compared to patients who discontinue or terminate OMT (11, 12). Nevertheless, national settings with differences in OMT coverage, clinical practice, and OMT medication modality on the one hand, and individual factors such as age and sex on the other, may be associated with differences in treatment outcomes including all-cause and overdose mortality rates (7).

Methadone and buprenorphine are currently the most widely used medications in OMT in Europe and elsewhere (13). Currently, in Europe, methadone is prescribed to 61% of patients in OMT, buprenorphine to 37%, and other types of medications including slow-release oral morphine to less than 4% (14). While methadone has been used in OMT for nearly five decades in Denmark (9) and over three decades in Czechia (15), buprenorphine first became available in 1999 in Denmark (16) and in 2000 in Czechia (15). Buprenorphine with naloxone was available a few years later (7). As opposed to the full mu-agonist methadone, buprenorphine is a partial agonist and was therefore introduced as a safety measure (17, 18). Naloxone, a non-selective opiate antagonist, in combination with buprenorphine was introduced as a strong deterrent to parenteral use, nevertheless, the expectations we not necessarily met (19). Previous studies showed that the use of buprenorphine in OMT was associated with a lower risk of both all-cause and overdose mortality compared to methadone (20), and recent systematic reviews and meta-analyses suggest lower all-cause mortality and overdose mortality risk related to buprenorphine both in and out OMT. However, this field lacks long-term, nation-wide studies, in particular those that include buprenorphine with naloxone.

Nation-wide OMT registers with detailed information on an individual level about time of enrolment in treatment, discontinuation, re-entry, and termination of OMT medication exist in Czechia and Denmark (21), and may be linked to information on time of death and causes of death obtained from nation-wide mortality registries. Furthermore, these two countries represent distinct treatment settings. Denmark adopts a more liberal and inclusive approach, whereas Czechia employs a more stringent treatment framework (9, 15, 22, 23). Examining mortality rates within and outside of OMT for each country individually can yield valuable insights for shaping clinical approaches.

The aim of this study was to investigate mortality rates among OMT patients during periods of both being in and out of OMT, within two countries that represent distinct treatment approaches. Specifically, within each of the two national OMT cohorts, we aimed to investigate the crude mortality rates (CMR) related to: (i) treatment status (episodes in and out of OMT); (ii) sex; (iii) cause of death (overdose or non-overdose); and (iv) OMT medication modality (methadone, buprenorphine, or buprenorphine with naloxone).

## Methods

### Study design

We used a prospective cohort approach linking the data sources by using the unique civil registration number assigned to all residents in both countries (21).

### OMT setting

#### Czechia

OMT has been available as standard treatment in Czechia since 2000. The procedures for OMT are defined in the Standards for Substitution Treatment (24).

In 2017, the OMT patient rate in Czechia was 0.6 per 1,000 population (7). OMT medication can be prescribed by any physician regardless of specialization, but under a strict prescription regime applied for controlled substances. None of the types of OMT medications are formally preferred in clinical guidelines. Buprenorphine is a widespread medication since methadone is available as magistral medicine only in 12 specialized treatment centres. Approximately 50 OMT providers, predominantly general psychiatry or general practitioner practices, are officially registered for the use of buprenorphine (24). As opposed to methadone, buprenorphine was not covered by the national health insurance plan, and patients had to pay for their own OMT medication (25). Generally, OMT programmes (especially methadone programmes) apply strict rules regarding treatment compliance and illicit drug use, and urine testing is therefore an integral part of the treatment regime. Annually, approximately 500 OMT episodes (out of approximately 2,500 patients registered annually) are terminated, with half of them excluded from treatment due to non-adherence (22).

#### Denmark

Denmark was one of the first countries in Europe that introduced OMT (in 1970), and one of the first countries that focused on achieving high treatment coverage and retention through a more inclusive approach. In 2017, Denmark had an OMT patient rate of 1.7 per 1,000 population (7). The increased accessibility of OMT progressed throughout the 1990s and 2000s. It has been characterized by a great number of enrolled patients with OUD, one of the highest prescription rates of methadone in Europe (26), and more liberal control measures with regards to concurrent use of alcohol and other drugs, as these measures were regarded as potential barriers to treatment uptake and retention (26). To facilitate greater inclusion, supervised intake of OMT medication, urine testing, and other safety measures have been minimized. Further, compared to many other countries, patients receiving OMT in Denmark often have more influence on choice of medication and doses, and number of take-home doses are often higher compared with many other countries (26). However, the individual choice and self-management may at times be challenged in clinical settings in order to adhere to the overall OMT guidelines (27). In 2008 (revised in 2017), the Danish Health Authority published the first guidelines on medications used as part of OMT and emphasized that buprenorphine with naloxone should be used as the drug of first choice for people with OUD who had not previously been enrolled in OMT (28). Since then, prescribing buprenorphine with naloxone to OMT patients has been prioritized, although older patients in particular have expressed their preferences for methadone over buprenorphine (29).

### Study population and study period

In Czechia, we included data from 1,580 females and 3,637 males who had been prescribed OMT medication at any time during the study period from January 1st 2000 to December 31st 2019.

In Denmark, we included 2,078 females and 6,387 males who had been prescribed OMT medication during the study period at any time from January 2nd 2007 to December 31st 2018. A total of 22 OMT patients who died during the study period were excluded from the analysis because of missing data on either cause or date of death.

### Data sources

In Czechia and Denmark, both data on exposure (OMT treatment) and outcome (death) were retrieved from national registers (20). Reporting to these registers is mandatory for the relevant professionals in both countries. A personal identification number is registered for each record, which enables linking data from various health registers at the individual level.

Czechia (21):

*The national registry of addiction treatment*

The national registry of addiction treatment (NRAT) contains information on patients in OMT, including diagnoses, OMT treatment details, and basic socio-demographic data of all patients.

*The information system on deaths*

The information system on deaths (ISZEM) is a general mortality register, which includes information on deaths of individuals with permanent or long-term residence in Czechia. The data is based on the information provided in the death certificate. The ISZEM contains the underlying and contributing causes of death based on ICD-10 codes. The data have been available in electronic form since 1994.

Denmark (21):

*The Danish registry of drug abusers undergoing treatment*

The registry contains information on individuals enrolled in treatment of drug use disorders due to illicit drug use since 1996. The register contains brief socio-demographic information, information on past year drug use, and dates of admission and discharge, and provides information to the european monitoring centre for drugs and drug addiction (EMCDDA) treatment demand indicator.

*Cause of death registry*

Dates and causes of death were identified using the general mortality register (30) that includes sex, age, and the underlying cause and contributing causes of death based on the ICD-10.

### Operationalization of OMT

**Figure 1:**
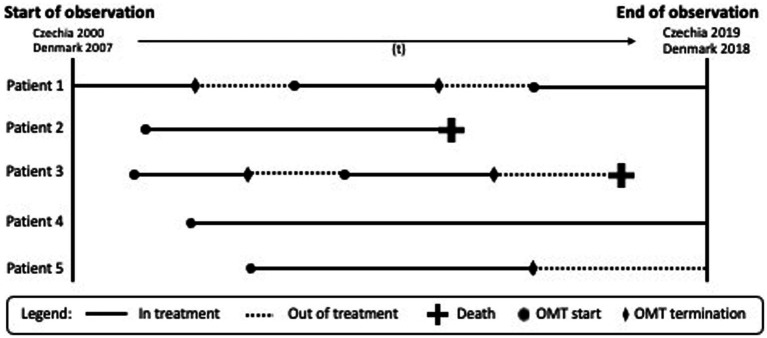
Examples of scenarios of patient trajectories in the opioid maintenance treatment (OMT) used in this study.

OMT patients were classified as *in treatment* or *out of treatment*. *In treatment* refers to the period that the individual patient was enrolled in OMT and was eligible to receive OMT medication. *Out of treatment* refers to the period that the individual patient was in between OMT treatment episodes or between the end of OMT episode and the end of follow-up if no new treatment episode was initiated (Figure 1).

### Analytic strategy

Statistical procedures included descriptive analyses of both study populations. The crude mortality rates (CMR) and 95% confidence intervals (CI) were calculated as the number of deaths per 1,000 person years (PY) in treatment and out of treatment (31) (see Figure 1). We calculated the time in treatment as the sum of days between treatment start and stop or the end of follow-up (i.e., death or end of observation). Time out of treatment was calculated as the sum of days between discharge from the treatment to the day of enrolment in a new treatment episode or from the treatment stop to the end of follow-up (i.e., death or end of observation).

Based on EMCDDA selection B, overdose was defined as deaths, where the underlying cause was mental and behavioural disorders caused by illicit drug use (dg. F11–F19 without F13, F17 and F18), or accidental, intentional, or undetermined illicit drug poisoning, i.e., a combination of the mechanism of death listed under X or Y with the diagnoses for substance poisoning (dg. T40 and T43.6) (32).

The direct standardization method using EU-27 + EFTA standard population 2011–2030 was employed to standardize for age (33).

The study period was 2000–2019 in Czechia and 2007–2018 in Denmark. Therefore, we performed sensitivity analysis on in treatment and out of treatment CMR in Czechia for the same period as in Denmark (2007–2018).

Statistical analyses were performed using SPSS (version 24) (34) and Stata (version 16) (35).

#### Ethics statement

Approval from the Ethics committee in Czechia (no. 36/19GrantAZVVES20201.LFUK) has been obtained. In Denmark, ethical evaluation of studies utilizing quality assurance data is not required by law but was approved by the data authority (Journal number: 2013540288, updated March 13th, 2020). No human studies are presented in this manuscript.

## Results

In Czechia, 1,580 (30.3%) out of 5,219 OMT patients were females, while 2,078 (24.5%) out of 8,465 OMT patients were females in Denmark (Table 1). The average age at first enrolment in OMT in study periods was more than 10 years lower in the Czech cohort compared with the Danish cohort. In Czechia, the proportion of patients who received methadone or buprenorphine as the initial medication was almost the same (38.4 and 38.7%, respectively), while 22.9% received buprenorphine with naloxone. In Denmark, 79.5% of patients initiated OMT with methadone.

**Table 1:** Characteristic of the study population.

	Czechia	Denmark
**Number of persons (who initiated OMT), *n*** ^**a**^	5,219	8,465
Female, *n* (%)	1,580 (30.3)	2078 (24.5)
Male, *n* (%)	3,637 (69.7)	6,387 (75.5)
**Age at treatment start in study period**		
Female, mean, median	27.3, 26	40.2, 40
Male, mean, median	29.8, 29	39.8, 39
**Medication at first admission in study period**		
methadone, *n* (%)	2003 (38.4)	6,731 (79.5)
buprenorphine, *n* (%)	2020 (38.7)	1,178 (13.9)
buprenorphine with naloxone, *n* (%)	1,196 (22.9)	556 (6.6)
**Multiple episodes, *n* (%)**	2,687 (51.5)	3,960 (46.8)

Opioid maintenance treatment (OMT) during 2000–2019 (*N* = 5,219)^a^ in Czechia. OMT during 2007–2018 (*N* = 8,465) in Denmark.

^a^No information on sex for two persons in Czechia.

In Czechia, 8.1% (421) of OMT patients (5,219) died during the study period (Table 2). In Denmark, 18.0% (1,526) of OMT patients (8,465) died during the study period.

**Table 2:** Characteristic of study population.

	Czechia			Denmark			
	Died from overdose (*n* = 47)	Died from non-overdose (*n* = 374)	Alive (*n* = 4,798)^a^	Died from overdose (*n* = 399)	Died from non-overdose (*n* = 1,105)	Died from unknown causes (*n* = 22)	Alive (*n* = 6,939)
**Age at death: mean, median**	35.4, 34	38.1, 37	NR	42.7, 43	49.1, 50	44.1, 46	NR
**Sex, *n* (%)**							
Female	6 (12.8)	77 (20.5)	1,497 (31.2)	81 (20.3)	276 (25.0)	*E*	1720 (24.8)
Male	41 (87.2)	297 (79.4)	3,299 (68.6)	318 (79.7)	829 (75.0)	*E*	5,219 (75.2)
**Medication at first admission**							
methadone	*E*	31 (8.3)	1975 (41.2)	371 (93.0)	1,038 (93.9)	*E*	5,114 (73.7)
buprenorphine	8 (17.0)	42 (11.2)	1917 (40.0)	15 (3.8)	38 (3.4)	*E*	1,306 (18.8)
buprenorphine with naloxone	*E*	*E*	1,174 (24.5)	13 (3.3)	29 (2.6)	*E*	519 (7.5)
**Multiple episodes, *n* (%)**	29 (61.7)	184 (49.2)	2,475 (51.6)	220 (55.1)	561 (50.8)	12 (54.5)	3,166 (45.6)

Causes of death specific to age, sex, opioid maintenance treatment medication, and treatment episodes in patients in opioid maintenance treatment in Czechia and Denmark.

^a^Two patients of unknown sex were not included in the sex-stratified analysis.

E (Ethics) represents the number of patients fewer than 5 that may not be displayed due to general data protection rules (GDPR). Alternatively, it represents the number that could enable the calculation of the count of patients fewer than 5.

NR, not relevant.

In Czechia, a quarter of all deaths occurred while in treatment, the CMR was 4.3/1,000 PY (females 3.0/1,000 PY, males 4.9/1,000 PY), the all-cause CMR out of treatment was higher (10.8/1,000 PY; females 6.7/1,000 PY, male 12.6/1,000 PY) (Table 3). The proportion of overdose deaths in treatment was 11.3% with an overdose-specific CMR of 0.5/1,000 PY. Overall, there were less than five poisoning by methadone (T40.3) overdose deaths among OMT patients.

**Table 3:** Crude mortality rate (CMR) per 1,000 person-years and 95% confidence interval (CI) in treatment and out of treatment in Czechia and in Denmark.

	Czechia						Denmark					
	**In treatment**			**Out of treatment**			**In treatment**			**Out of treatment**		
	***n***	**PY**	**CMR (CI)**	***n***	**PY**	**CMR (CI)**	***n***	**PY**	**CMR (CI)**	***n***	**PY**	**CMR (CI)**
Overdose	12	24,495	0.5 (0.2–0.8)	35	29,246	1.2 (0.8–1.6)	269	36,886	7.3 (6.5–8.2)	130	18,556	7.0 (5.9–8.3)
Non-overdose	94	24,495	3.8 (3.1–4.6)	280	29,246	9.6 (8.5–10.7)	712	36,886	19.3 (18.0–20.8)	393	18,556	21.2 (19.2–23.4)
All causes*	**106**	**24,495**	**4.3 (3.5–5.2)**	**315**	**29,246**	**10.8 (9.6–12.0)**	**981**	36,886	**26.6 (25.0–28.3)**	**523**	18,556	**28.2 (25.9–30.7)**
**Female**							**Female**					
Overdose	E	–	–	5	9,048	0.6 (0.1–1.0)	61	9,636	6.3 (4.9–8.1)	20	4,124	4.9 (3.1–7.5)
Non-overdose	E	–	2.9 (1.6–4.1)	56	9,048	6.2 (4.6–7.8)	182	9,636	18.9 (16.4–21.8)	94	4,124	22.8 (18.7–27.8)
**All causes**	**22**	**7,321**	**3.0 (1.7–4.3)**	**61**	**9,048**	**6.7 (5.0–8.4)**	**243**	9,636	**25.2 (22.3–28.5)**	**114**	4,124	**27.6 (23.1–33.1)**
**Male**							**Male**					
Overdose	11	17,162	0.6 (0.3–1.0)	30	20,179	1.5 (1.0–2.0)	208	27,250	7.6 (6.7–8.7)	110	14,432	7.6 (6.3–9.2)
Non-overdose	73	17,162	4.3 (3.3–5.2)	224	20,179	11.1 (9.6–12.6)	530	27,250	19.5 (17.9–21.2)	299	14,432	20.7 (18.5–23.2)
**All causes**	**84**	**17,162**	**4.9 (3.8–5.9)**	**254**	**20,179**	**12.6 (11.0–14.1)**	**738**	27,250	**27.1 (25.2–29.1)**	**409**	14,432	**28.3 (25.8–31.2)**

All cause, overdose, and non-overdose death stratified on sex.

*Two patients of unknown sex were not included in the sex-stratified analysis.

E (Ethics) represents the number of patients fewer than 5 that may not be displayed due to general data protection rules (GDPR). Alternatively, it represents the number that could enable the calculation of the count of patients fewer than 5.

In Denmark, 65.2% of deaths occurred while in treatment. The all-cause CMR in treatment was 26.6/1,000 PY, and out of treatment 28.2/1,000 PY. The proportion of overdose deaths in treatment was 27.4% while overdose-specific CMR was 7.3/1,000 PY. Poisoning by methadone (T40.3) in overdose deaths was found in 63.9% of female and 62.0% of male OMT patients while in treatment as opposed to 40.0% of females and 35.5% of males out of treatment.

In Czechia, all-cause CMR in treatment was quite similar across the types of OMT medication; methadone 3.8/1,000 PY (CI 95% 2.5–5.0), buprenorphine 4.9/1,000 PY (3.5–6.2), buprenorphine with naloxone 4.3/1,000 PY (2.5–6.0).

In Denmark, all-cause CMR in treatment specific to types of OMT medication was the highest for methadone 29.6/1,000 PY (27.8–31.5) and comparable for buprenorphine 8.2/1,000 PY (5.7–11.9) and buprenorphine with naloxone 10.8/1,000 PY (7.2–16.4).

Age-standardized mortality rate for OMT patients in Czechia and Denmark was 9.7/1,000 PY and 29.8/1,000 PY, respectively. Age-standardized overdose mortality rate for OMT patients in Czechia was 0.6/1,000 PY and 5.2/1,000 PY for Denmark.

Sensitivity analysis on CMR in Czechia for the same period as in Denmark (2007–2018) showed similar results as for the longer observation period (2000–2019). In treatment, the CMR was 4.6/1,000 PY (females 2.7/1,000 PY, males 5.4/1,000 PY), while the all-cause CMR out of treatment was (11.6/1,000 PY; females 7.2/1,000 PY, males 13.5/1,000 PY) (Supplementary Table S1).

## Discussion

In this large cohort study involving over 13,000 patients ever enrolled in OMT, we found higher mortality rates both in and out of OMT among patients in Denmark than in Czechia. The proportion of overdose deaths while in OMT was nearly 15 times higher among Danish patients than their Czech counterparts. In contrast to previous studies (1), the all-cause mortality rate in Denmark was similar for patients while in OMT as compared to patients while out of OMT. In Czechia, CMR remained consistent across all three OMT medication modalities (methadone, buprenorphine, and buprenorphine with naloxone), while in Denmark, the highest CMR was associated with methadone usage.

The key finding of this study was that the elevated CMR within the Danish OMT cohort was comparable to the rates observed outside of OMT. This pattern held true for both overdose and non-overdose deaths, as well as across both female and male participants. In Czechia, the all-cause mortality rate was more than two times higher out of treatment than in treatment, as has been observed in multiple other settings, such as Norway and Australia (36). Out of treatment mortality was higher for males than females, while the sex difference was not as prominent in treatment. In Denmark, sex differences in mortality were not so pronounced either in or out of treatment.

The population of OMT patients in Denmark was approximately 10 years older on average at the time of enrolment in OMT compared with Czechia. This difference could explain the higher mortality in Denmark. However, even after age standardization of crude mortality rates the differences between mortality rates remained.

The significant differences in overdose deaths between Czechia and Denmark may be attributed to differences in OMT systems. The OMT settings differ markedly in Czechia and Denmark, with the Danish system being more inclusive (9). There may be positive benefits of this more liberal approach, such as the inclusion of a larger proportion of people with OUD who may be severely dependent or less likely to abstain from use of illicit drugs while in OMT as well as an overall higher treatment participation rate. The rate of patients receiving OMT in Denmark was nearly three times higher than that in Czechia (per 1,000 population in 2017) (7). Nevertheless, the unexpectedly elevated rates of both all-cause and overdose mortality rates among Danish OMT patients prompt inquiries into the efficacy of the treatment approach.

Alongside Denmark’s more liberal and inclusive OMT approach, methadone stands out as the most commonly prescribed OMT medication in the country. Patients treated with methadone had both higher all-cause mortality rates in Denmark compared with patients treated with buprenorphine or buprenorphine with naloxone. The pharmacological properties of methadone, a full mu-opioid agonist, might explain at least some of the high overdose mortality rate in patients treated with methadone. Poisoning by methadone in the overdose death group was high both while in treatment (over 60%) and while out of treatment (nearly 40%) in Denmark. The inclusivity of the OMT system in Denmark, along with its minimum requirements for supervised intake of OMT medication, urine testing, or other safety measures (9, 26) combined with the pharmacological properties of methadone, might be linked to this finding. In Denmark, OMT medication can be administered at OMT clinics or dispensed at pharmacies where there is less supervision of intake. A recent Danish cohort study on dispensing of OMT medication at pharmacies highlighted the risks associated with such a practice in the Danish context (23). The study found that after having methadone or buprenorphine dispensed at a pharmacy, adverse outcomes, including all-cause mortality, increased. Thus, this practice may also have contributed to the high in-treatment mortality found in Denmark. In this regard, naloxone, with its pharmacological properties (37), may help to effectively respond to overdoses and overdose deaths (38, 39).

Our findings indicate that there is a potential for improvement in clinical practices by applying a systematic evaluation and monitoring of mortality risk, increased involvement of psychosocial services, and enhanced safety measures to manage overdoses and other health risks. The differences between the Czech and Danish clinical approaches are substantial; one approach focuses more on safety and restrictions while the other focuses more on inclusion and reduced control measures (9, 22, 23). It may be that many patients who relapse to harmful drug use are excluded from treatment in Czechia. Thus, periods of relapse are likely to be more prevalent in treatment in Denmark and more prevalent while out of treatment in Czechia. Expelling patients from treatment while they are experiencing a symptom-intense period or not responding clinically to these symptom-intense situations while patients are in treatment are suboptimal treatment approaches. Thus, the key to lower mortality rates in OMT patients may be found in a more balanced liberalization of the treatment system that both seeks to include people with OUD, despite unstable adherence and periodic excesses in substance use, and to prioritize clinically safe treatment practices, including closer clinical patient monitoring. For example, the move toward more inclusive OMT in Norway has been achieved without an increase in mortality rates (36).

We should not think of the three main features in OMT - access, safety, and quality - as mutually exclusive, but rather as important dimensions of treatment that must be addressed and optimized. This would indeed be in accordance with the Dole, Nyswander, and Kreek recommendations for OMT from the 1960s (40).

### Strengths and limitations

This study was unique because we were able to apply similar analytic approaches to study differences in mortality of OMT patients based on a comparison of nation-wide patient cohorts from two countries with different OMT settings. Further, we were able to study mortality rates among those in and out of OMT and while using different OMT medications (methadone, buprenorphine, and buprenorphine with naloxone) by causes of death (overdose, non-overdose), age, and sex. Information about OMT patients was drawn from the nation-wide health and population-based registers in Czechia and Denmark, minimizing selection and information bias. Finally, yet importantly, the information from the registers includes the specific dates for entering, terminating, and re-entering OMT on an individual patient level. Thus, the individual patient follow-up can be precisely calculated with respect to CMR. Additionally, this study provided new information on mortality rates linked to buprenorphine with naloxone.

Our study also had limitations. The main limitation is that some important clinical information was not included in this study (41, 42), including concurrent legal and illegal substance use while in or out of treatment, some demographic and socio-cultural variables in the dataset, or data on OMT medication doses and medication compliance. In general, data from the Czech and Danish registers are highly compatible. However, some types of data were missing. For example, information about 34 Danish patients (29 females) who died and could not be included in the CMR analysis may have resulted in an underestimation of mortality rate in Denmark, especially for females. These individuals were not included in the analyses because we did not have sufficient data on, for instance, cause of death or date of death. In addition, there might be differences in coding of overdose deaths; in Czechia, overdoses may be coded as accidents or self-harm to a greater extent than in Denmark. Finally, the study period in Denmark was between 2007 and 2018 with considerable proportion of patients enrolled in OMT before 2007. This may have resulted in higher age of Danish patients at treatment start in the study period.

## Conclusion

We observed significant differences in all-cause and overdose mortality rates in patients while in or out of OMT in Czechia and Denmark. Higher mortality rates were observed across the different types of OMT medication as well as in both sexes in the Danish cohort compared to the Czech cohort. These differences may be attributed to the clinical characteristics in both patient populations and to differences in clinical practice in OMT in the two countries. The findings are indicative and call for more studies on risk factors related to premature mortality as well as risk management.

In Denmark, provision of liberal access to OMT did not necessarily lead to expected reduction in overdose deaths while in treatment. In Czechia, more inclusive clinical practice might lead to higher OMT coverage. Perhaps, a better balance of patient safety and access to OMT would lead to a reduced risk of overdose deaths.

Our study shows how important yet challenging it is to extrapolate mortality estimates from one setting to another and to generalize findings across settings and populations. The findings contribute to the discussion about OMT management and may be of interest to practitioners as well as policy and decision makers when balancing the safety and accessibility of OMT.

## Data availability statement

The data analyzed in this study is subject to the following licenses/restrictions: this project uses third-party data derived from State government registries and databases, which are ultimately governed by their ethics committees and data custodians. Thus, any requests to share these data will be subject to formal approval from each data source used in this project. Czechia: Requests for data sharing/case pooling may be directed to the corresponding author RG on email: roman.gabrhelik@lf1.cuni.cz. Denmark: Requests for data sharing/case pooling may be directed to mh.crf@psy.au.dk. Requests to access these datasets should be directed to roman.gabrhelik@lf1.cuni.cz; mh.crf@psy.au.dk.

## Ethics statement

The studies involving humans were approved by the study has been reviewed and approved by the responsible ethics committee (the respective reference numbers are: 36/19GrantAZVVES20201.LFUK). For Denmark, the project is not under the legislation for ethical evaluation, since it is based entirely on administrative data, but the data has been approved by the data authority (Journal number: 2013540288, updated March 13, 2020). The studies were conducted in accordance with the local legislation and institutional requirements. Written informed consent for participation was not required from the participants or the participants’ legal guardians/next of kin in accordance with the national legislation and institutional requirements.

## Author contributions

SS, RG, and MoH designed the study. SS and BN analyzed the Czech data. MoH and AS analyzed the Danish data. All authors contributed to the interpretation of data, refinement of the paper, read, and approved the final version of the manuscript.
