# Supplementary material for: Large variations in all-cause and overdose mortality among >13,000 patients in and out of opioid maintenance treatment in different settings: a comparative registry linkage study
Source: Front Public Health. 2023 Sep 22;11:1179763. doi: 10.3389/fpubh.2023.1179763 (PMC10558053; doi:10.3389/fpubh.2023.1179763)
Supplement: Supplementary file 1 [file Table_1.DOCX]

Supplementary Table 1 Crude mortality rate (CMR) per 1000 person-years and 95% confidence interval (CI) during treatment with methadone, buprenorphine, or buprenorphine with naloxone in Czechia and Denmark.

| **Czechia** | **Methadone** | | | **Buprenorphine** | | | **Buprenorphine/naloxone** | | |
| --- | --- | --- | --- | --- | --- | --- | --- | --- | --- |
|  | **n** | **PY** | **CMR (CI)** | **n** | **PY** | **CMR (CI)** | **n** | **PY** | **CMR (CI)** |
| Overdose | E | - | - | 8 | 10 289 | 0.8 (0.2-1.3) | E | - | - |
| Non-overdose | E | - | 3.4 (2.2-4.6) | 42 | 10 289 | 4.1 (2.8-5.3) | E | - | 4.1 (2.3-5.8) |
| **All causes** | **34** | **9 036** | **3.8 (2.5-5.0)** | **50** | **10 289** | **4.9 (3.5-6.2)** | **22** | **5 171** | **4.3 (2.5-6.0)** |
|  | | | | | | |  | | |
| **Denmark** |  |  |  |  |  |  |  |  |  |
| Overdose | 255 | 31452 | 8.1 (7.2-9.2) | E | - | - | E | - | - |
| Non-overdose | 676 | 31452 | 21.5 (20.0-23.2) | E | - | - | E | - | - |
| **All causes** | **931** | **31452** | **29.6 (27.8-31.5)** | **28** | **3 404** | **8.2 (5.7-11.9)** | **22** | **2 030** | **10.8 (7.2-16.4)** |
|  | | | | | | |  | | |

E (Ethics) represents the number of patients fewer than 5 that may not be displayed due to general data protection rules (GDPR). Alternatively, it represents the number that could enable the calculation of the count of patients fewer than 5.
